# The Role of ERK Signaling in Experimental Autoimmune Encephalomyelitis

**DOI:** 10.3390/ijms18091990

**Published:** 2017-09-15

**Authors:** Katharina Birkner, Beatrice Wasser, Julia Loos, Alexander Plotnikov, Rony Seger, Frauke Zipp, Esther Witsch, Stefan Bittner

**Affiliations:** 1 Department of Neurology, Focus Program Translational Neuroscience (FTN) and Immunotherapy (FZI), Rhine Main Neuroscience Network (rmn2), University Medical Center of the Johannes Gutenberg University Mainz, Langenbeckstr. 1, 55131 Mainz, Germany; katharina.birkner@unimedizin-mainz.de (K.B.); beatrice.wasser@unimedizin-mainz.de (B.W.); julia.loos@unimedizin-mainz.de (J.L.); frauke.zipp@unimedizin-mainz.de (F.Z.); esther.witsch@gmail.com (E.W.); 2 The Nancy and Stephen Grand Israel National Center for Personalized Medicine, Weizmann Institute of Science, 234 Herzl Street, 7610001 Rehovot, Israel; alexander.plotnikov@weizmann.ac.il; 3 Department of Biological Regulation, Weizmann Institute of Science, 234 Herzl Street, 7610001 Rehovot, Israel; rony.seger@weizmann.ac.il

**Keywords:** T cells, ERK pathway, EPE peptide, experimental autoimmune encephalomyelitis, multiple sclerosis, cell signaling

## Abstract

Extracellular signal-regulated kinase (ERK) signaling plays a crucial role in regulating immune cell function and has been implicated in autoimmune disorders. To date, all commercially available inhibitors of ERK target upstream components, such as mitogen-activated protein (MAP) kinase/ERK kinase (MEKs), but not ERK itself. Here, we directly inhibit nuclear ERK translocation by a novel pharmacological approach (Glu-Pro-Glu (EPE) peptide), leading to an increase in cytosolic ERK phosphorylation during T helper (Th)17 cell differentiation. This was accompanied by diminished secretion of granulocyte-macrophage colony-stimulating factor (GM-CSF), a cytokine influencing the encephalitogenicity of Th17 cells. Neither the production of the cytokine interleukin (IL)-17 nor the proliferation rate of T cells was affected by the EPE peptide. The in vivo effects of ERK inhibition were challenged in two independent variants of experimental autoimmune encephalomyelitis (EAE), an animal model of multiple sclerosis (MS). Overall, ERK inhibition had only a very minor impact on the clinical disease course of EAE. This indicates that while ERK translocation might promote encephalitogenicity in T cells in vitro by facilitating GM-CSF production, this effect is overcome in more complex in vivo animal models of central nervous system (CNS) autoimmunity.

## 1. Introduction

Extracellular signal-regulated kinase (ERK) signaling is known to play a crucial role in regulating cellular proliferation, differentiation, and survival [1]. Aberrant ERK signaling is involved in carcinogenesis [2], and attempts to target the ERK cascade have shown therapeutic potential in the fight against cancer [3,4,5]. Furthermore, recent studies suggest a role of the ERK cascade in the innate immune system and in autoimmune responses [6]. The mitogen-activated protein (MAP) kinase/ERK kinase (MEK) components upstream of ERK and ERK itself regulate multiple facets of the immune system [7,8,9]. Multiple pieces of evidence point to the importance of ERK signaling in T cells, starting from signal transduction from the T cell receptor (TCR) to the G protein Ras and further to downstream components of the kinase cascade, namely, Raf, MEK, and ERK. In peripheral T cells, signaling via Ras is needed for TCR-initiated ERK activation [10,11]. T cells and macrophages showed phosphorylation of the MAP kinases ERK, JNK, and p38, and it was suggested that phosphorylated MAP kinases are important in the acute initiation of experimental autoimmune encephalomyelitis (EAE), an animal model for human multiple sclerosis (MS) [7]. In the EAE model, pharmacological inhibition of the MEK component upstream of the ERK ameliorated disease severity. Inhibition of the ERK cascade leading to suppressed interleukin (IL)-23 and IL-1β secretion by dendritic cells (DCs) was identified as the underlying route, subsequently leading to reduced IL-17 production by proinflammatory T helper (Th)17 cells [8]. In contrast, our lab has previously shown that ERK1-deficient dendritic cells are more potent at priming a T cell response [9]. Studies of ERK activity in peripheral T cells in rheumatoid arthritis patients and in a mouse model of this disease showed augmented ERK activity in hyperactive T cells [12]. Most of the known potentially therapeutic inhibitors of the ERK cascade target up-stream components, such as growth factor receptors, Raf kinases, and MEKs, and only one inhibitor, SCH772984, targets ERK itself [13]. It became clear that the low efficacy of these drugs is mainly due to the prevention of negative feedback loops initiated by the ERK cascade [5,14,15]. Recently, a novel motif within ERK, called nuclear translocation signal (NTS), was identified. In resting T cells, most of the ERK1/2 molecule is localized in the cytoplasm connected to a variety of cytosolic proteins such as cytoskeletal components and scaffold proteins as their anchoring points [16]. Upon activation of ERK1/2, it detaches from its anchor and translocates into the nucleus involving a TEY phosphorylation-dependent conformational change [17]. The phosphorylation of the thereby exposed NTS then allows for binding to Importin-7 (Imp7), and consequently induces the nuclear translocation of these kinases. The myristoylated, NTS-derived phosphomimetic peptide (EPE peptide) inhibits the interaction of Imp7 with ERK1/2, and consequently the nuclear translocation of the latter [18]. Stimulation-induced phosphorylation resulted in its interaction with Imp7 and nuclear translocation of ERK [2,19]. Given the dominant role of the nuclear function of ERK in mediating proliferation and differentiation, prevention of the nuclear translocation of ERK should inhibit proliferation without affecting the initiation of the negative feedback loops. Indeed, studies using peptide inhibitors of translocation prevented nuclear entry of ERKs and resulted in slower growth of various cells without affecting the AKT pathway [18,20]. However, the potential of the inhibition of ERK translocation in autoimmune neuroinflammation has not been tested so far.

In this study, we found that inhibition of ERK translocation by the EPE peptide decreases proliferation of splenocytes but not of autoreactive Th17 cells. We show that blockade of the ERK cascade with the MEK inhibitor UO126 suppressed T cell proliferation rates. Th17 differentiation increased intracellular ERK phosphorylation. The secretion of the cytokine granulocyte-macrophage colony-stimulating factor (GM-CSF) was diminished by the EPE peptide. When tested in an animal model of MS, the inhibitory peptide EPE did not affect the outcome of EAE. We conclude that ERK translocation might promote encephalitogenicity in T cells in vitro by facilitating GM-CSF production, but this effect is overcome in the more complex in vivo animal model of MS.

## 2. Results

### 2.1. ERK Translocation Promotes Encephalitogenicity in T Cells by Facilitating GM-CSF Production

**Figure 1:**
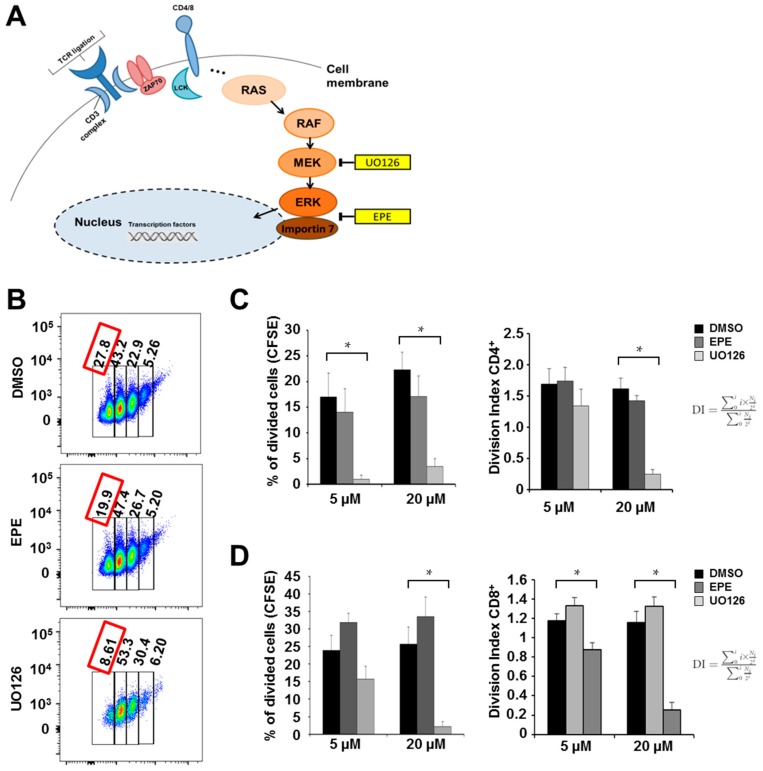

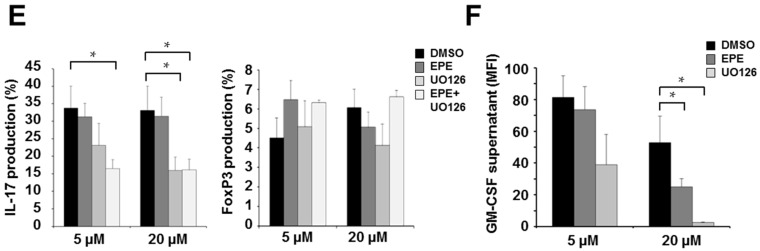
Inhibition of extracellular signal-regulated kinase (ERK) decreased the expression of granulocyte-macrophage colony-stimulating factor (GM-CSF) but not interleukin (IL)-17 upon T helper (Th)17 cell differentiation. (**A**) Schematic illustration of the mitogen-activated protein kinase (MEK)-ERK pathway and illustration of pharmacological inhibitors; (**B,C**) CFSE-labeled CD4^+^ T cells from the spleen of C57BL/6x2d2 mice were stimulated with antibodies to CD3 (3 μg/mL) and CD28 (2.5 μg/mL) and treated with EPE (5 μM, 20 μM) and UO126 (5 μM, 20 μM) for 72 h. Bar charts show the percentage of divided cells that reached four proliferation cycles and represent six independent experiments. Five micromolars (5 μM) EPE vs. DMSO, *p* = 0.66, 20 μM EPE vs. DMSO, *p* = 0.34. Division index (DI) was calculated according to FlowJo defined by the average number of divisions for all cells in the culture [22]. Five micromolars (5 μM) EPE vs. DMSO, *p* = 0.89, 20 μM EPE vs. DMSO, *p* = 0.35, 5 μM UO126 vs. DMSO, *p* = 0.36; (**D**) CFSE-labeled CD8^+^ T cells from the spleen of C57BL mice were stimulated with antibodies to CD3 (3 μg/mL) and CD28 (2.5 μg/mL) and treated with EPE (5 μM, 20 μM) and UO126 (5 M, 20 μM) for 72 h (control = DMSO). Bar charts show the percentage of divided cells that reached four proliferation cycles and represent six independent experiments. Five micromolars (5 μM) EPE vs. DMSO, *p* = 0.15, 20 μM EPE vs. DMSO, *p* = 0.32, 5 μM UO126 vs. DMSO, *p* = 0.18. Division index was calculated according to FlowJo defined by the average number of divisions for all cells in the culture [22]. 5 μM EPE vs. DMSO, *p* = 0.19, 20 μM EPE vs. DMSO, *p* = 0.29; (**E**) Naïve CD4^+^ T cells from C57BL/6x2d2 mice were stimulated with antibodies to CD3 (3 μg/mL) and CD28 (2.5 μg/mL) under Th17-promoting culture conditions and treated with EPE and UO126 for 72 h. On day three cells were stimulated again with phorbol 12-myristate 13-acetate (PMA) (1:200) and ionomycin (1:1000) for 4 h, harvested and stained for IL-17 (5 μM UO126 vs. DMSO, *p* = 0.24) and FoxP3 (5 μM EPE vs. DMSO, *p* = 0.2, 20 μM EPE vs. DMSO, *p* = 0.43, 5 μM EPE/ UO126 vs. DMSO, *p* = 0.13, 20 μM UO126 vs. DMSO, *p* = 0.21). Bar charts represent a summary of eight independent experiments; (**F**) Cell culture supernatants from five independent Th17 in vitro cultures were analyzed using a bead-based immunoassay measuring GM-CSF. Treating cells with either EPE or UO126 significantly decreased protein secretion of GM-CSF. All error bars show mean fluorescence intensity (MFI) and standard error of the mean (SEM). *p*-Values were obtained using unpaired student-*t*-test comparing two groups. * *p* < 0.05.

Activation and proliferation of CD4^+^ T helper cells is crucial for the onset and progression of EAE disease in mice, thus we first tested whether inhibition of the ERK cascade influences proliferation in peripheral CD4^+^ splenocytes (Figure 1A). It has been shown before that MEK inhibitors hinder ERK phosphorylation and restrict T lymphocyte function. Especially the MEK inhibitor U0126 was shown to impair proliferation and cytokine secretion of T lymphocytes [21]. Therefore, we measured the effects of U0126 and EPE on proliferation and cytokine production. CD4^+^ and CD8^+^ differentiated cells were labeled with carboxyfluorescein succinimidyl ester (CFSE) and stimulated with anti-CD3 and anti-CD28 in the presence of dimethyl sulfoxide (DMSO, control solvent), EPE peptide, or UO126 for 72 h. Measurement of proliferation using flow cytometry showed significant reduction in proliferation upon MEK inhibition. Treatment with the EPE peptide led to a slight, non-significant, decrease in proliferation in a concentration-dependent manner (Figure 1B,C). The proliferation of CD8 cells was unchanged after EPE treatment whereas MEK inhibition with UO126 inhibited CD8 cell proliferation (Figure 1D) significantly. Intracellular expression of the cytokine IL-17 and the transcription factor FoxP3, which indicate changes in T cell plasticity, were analyzed by flow cytometry. At all concentrations tested, the production of IL-17 was significantly reduced by MEK inhibition though not by EPE. At higher concentrations there was no synergistic effect achieved by EPE and UO126 treatment (Figure 1E). FoxP3 production was not found to be changed significantly (Figure 1E). Interestingly, secretion of GM-CSF, a cytokine influencing the encephalitogenicity of Th17 cells, was diminished by both EPE and UO126 (Figure 1F).

### 2.2. Differentiation Towards a Th17 Phenotype Increases ERK Phosphorylation with No Effect by EPE Peptide

**Figure 2:**
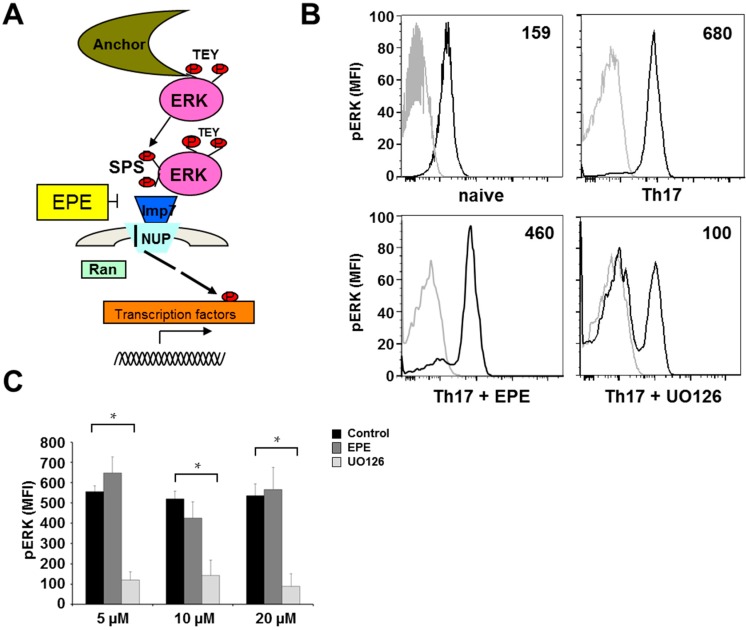
Treatment with UO126 but not EPE peptide ERK inhibits phosphoTEY-ERK expression in differentiating Th17 cells. (**A**) Schematic illustration of ERK pathway and ERK phosphorylation sites TEY and SPS [23,24]; (**B**) Histograms represent the pERK staining (black line) in comparison to the isotype staining (grey line) in Th17 cells developing 72 h under Th17-promoting culture conditions. Differentiation under Th17-promoting conditions increased the ERK phosphorylation in comparison to naïve cells; (**C**) Expression of the TEY pERK sequence is only inhibited after treatment with MEK inhibitor UO126. Bar charts represent a summary of five independent experiments; error bars show SEM. *p*-Values were obtained using unpaired student-t-test comparing two groups. * *p* < 0.05.

It is known that inhibition of upstream components of the cascade abolishes ERK activatory phosphorylation at its TEY motif [23,24], whereas inhibition of ERK’s nuclear translocation using EPE peptide should not affect phosphorylation of ERK-TEY (Figure 2A). To verify this, we carried out intracellular staining of the TEY phosphorylation site at the ERK molecule using an anti-phospho-TEY antibody and analyzed the signal by flow cytometry. ERK expression was significantly increased after 72 h under Th17-promoting culture conditions compared to naïve CD4^+^ T cells (Figure 2B). Treatment of cells during Th17 differentiation using U0126 inhibited ERK-TEY phosphorylation, whereas EPE peptide had no influence on the ERK-TEY phosphorylation (Figure 2C).

### 2.3. Inhibition with the EPE Peptide Has Only Discrete Influence on the Outcome in Two Different EAE Models

**Figure 3:**
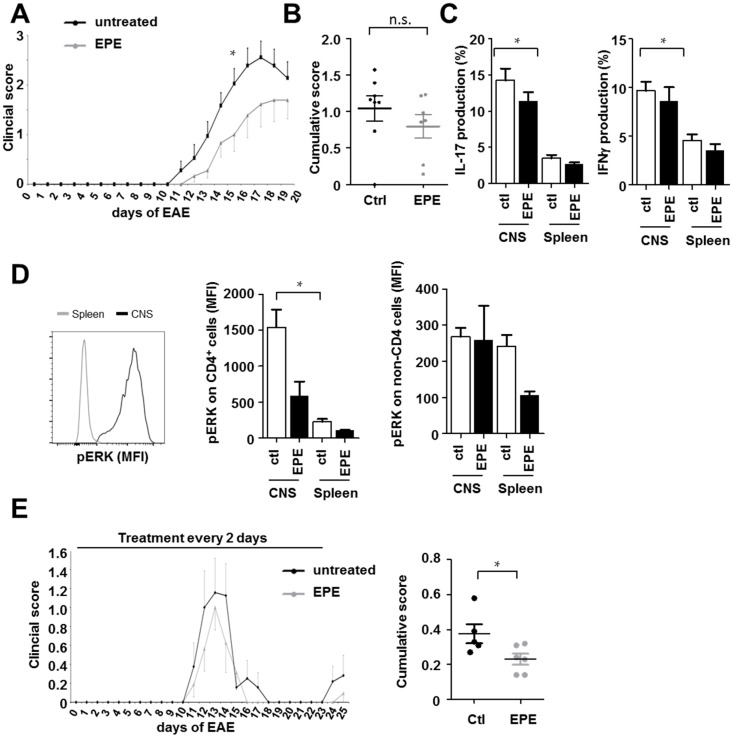
The inhibitory EPE peptide has only minor impact in EAE models in vivo. (**A**) Active EAE in C57BL/6 mice was induced by the injection of myelin oligodendrocyte glycoprotein (MOG)_35–55_/complete Freund's adjuvant (CFA) emulsion followed by pertussis toxin. EPE peptide was administered intraperitoneally (i.p.) two days before disease induction, and then every other day until day 18; (**B**) The cumulative score of all EAE animals treated with EPE and DMSO (as control) was assessed. On day 18 after EAE induction, leukocytes from the central nervous system (CNS) and splenocytes were stimulated ex vivo and stained for; (**C**) IL-17 and IFNγ; (**D**) pERK. Bar charts represent the mean percentage of nine mice and error bars show SEM (CD4^+^ cells: DMSO CNS vs. EPE CNS, *p* = 0.95 and DMSO Spleen vs. EPE Spleen, *p* = 0.09; non-CD4 cells DMSO Spleen vs. EPE Spleen, *p* = 0.06); (**E**) Active EAE was induced in female SJL mice via injection with murine PLP_139–151_. Intraperitoneal administration of DMSO as a solvent (*n* = 5) or EPE peptide (*n* = 6) started two days before immunization, and then every other day until day 20. EPE administration slightly reduced the EAE course; however, the cumulative score was significantly decreased by the treatment with EPE. Error bars show SEM. *p*-Values were obtained using unpaired student-*t*-test comparing two groups. * *p* < 0.05.

Previous studies showed that treatment with the MEK inhibitor U0126 ameliorated the course of active EAE when administered during disease induction as well as after disease onset [8]. C57BL/6 mice were actively immunized and treated intraperitoneally (i.p.) with EPE peptide two days before disease induction, and then every other day until day 18. Mice treated with the EPE peptide showed a non-significant trend towards ameliorated disease symptoms (Figure 3A,B, see also Table 1 for data on all EAE experiments). On day 18 after EAE induction, leukocytes from the central nervous system (CNS) and the spleen were isolated and stimulated ex vivo with PMA and ionomycin and analyzed for their cytokine profile. IL-17 and IFNγ production were both elevated in CNS-derived lymphocytes in comparison to those from the periphery (Figure 3C). Furthermore, TEY ERK phosphorylation was significantly higher in CD4^+^ cells from the CNS than in CD4^+^ cells from the periphery (Figure 3D). Cells from EPE peptide-treated mice showed no effect on IL-17 or IFNγ production (Figure 3C), or on the expression of phospho-ERK (Figure 3D). Thereafter, the effect of EPE was tested in a second model of EAE, namely proteolipid protein (PLP)-induced relapsing-remitting EAE in SJL mice. In this model, treatment with the EPE peptide showed almost no effect on EAE outcome when administered i.p., two days before disease induction, and again every other day until day 20 (Figure 3E, see also Table 1 for data on all EAE experiments). Mild disease amelioration lasted only until the end of the treatment protocol although the cumulative score at day 20 was significantly reduced (Figure 3E). Taken together, these results indicate that the inhibition of nuclear ERK translocation has only minor influence in complex in vivo models of CNS autoimmunity.

## 3. Discussion

Protein kinases are considered to be attractive drug targets and a number of chemicals are in clinical trials for the combat against cancer as well as autoimmune diseases such as rheumatoid arthritis [25]. In this study, we show that the secretion of granulocyte-macrophage colony-stimulating factor (GM-CSF) in Th17 cells was diminished by the inhibition of the nuclear ERK translocation with the EPE peptide. However, the outcome of EAE models in vivo was not ameliorated. We conclude that ERK translocation might promote encephalitogenicity in T cells in vitro, but that this effect is overcome by cytoplasmic ERK activation in more complex in vivo situations.

It has been reported that ERK^−/−^ mice have increased susceptibility to EAE [26], while its cellular mechanisms are incompletely understood. In this context, one has to keep in mind that phosphorylation of ERK is thought to play a pivotal role in a wide range of cellular activities, and a number of studies have demonstrated a major role for the components of the ERK cascade in the regulation of the innate and adaptive immune responses, though most of the studies only investigate the role of Th1 and Th2 cells [26,27,28,29]. All of these studies highlight the importance of ERKs as crucial regulators of Th1 and Th2 cell activation and effector cytokine expressions of IL-4 and IL-2 [27], but these studies did not investigate the phenotype of Th17 cells. In in vivo studies where MEK1/2 was inhibited, clinical signs of disease were ameliorated in EAE due to a negative regulator function of ERK1 on autoimmune Th1 responses, but T cell development of other subtypes such as Th17 cells was not influenced in vivo [8]. This might explain why targeting ERK functionality in Th1 cells might have a more pronounced impact than targeting Th17 cells in vivo. Given the current understanding that IL-23, rather than IL-12, is the critical cytokine for the establishment and persistence of inflammatory lesions in EAE [30,31,32], one study investigated the possibility of not targeting ERK signaling of Th17 cells directly. Instead, they made use of DCs from ERK1^−/−^ mice, which produce greater levels of IL-23, which then indirectly resulted in enhanced IL-17 production of T cells. However, they failed to detect any differences in IL-23 production of DCs from WT versus ERK^−/−^ mice, again indicating a disconnection between the significant effects of ERK1 deficiency on the induction of IL-2 and IL-10 and the only modest effects on EAE pathogenesis [26].

Another important point is that most of the published EAE studies target MEK components and inhibit upstream of ERK to ameliorate disease outcome, which then is accompanied by a reduced ability of T cells to produce IL-17 and IFNγ [7,8,9,26]. Thereby, we conclude that ERK itself, being downstream of MEK, does not play a pivotal role in Th17 cell-mediated pathogenicity in EAE. However, the approach to target nuclear ERK itself by using the inhibitory EPE peptide provided significant insight into this pathway, in comparison to several components targeting upstream of ERK. In this context, PD98059 and U0126 are directed against MEK1 and MEK2 [33,34]. MEK inhibitors such as PD0325901 [21] and PD98059 [35,36] have been shown to exhibit negative effects on T-lymphocyte proliferation and cytokine secretion and were thereby excluded for any experimental set ups.

Studying the animal model of MS, one has to keep in mind that the formation and maintenance of a functional blood brain barrier (BBB) plays a pivotal role and relies on a unique crosstalk of brain microvascular endothelial cells with their neighboring cells such as astrocytes, microglia and neurons. In mice suffering from EAE, encephalitogenic T cells breach the BBB which is associated with the severity of MS and little is known so far whether the BBB integrity in MS might be influenced by ERK-inhibition or the reduction of the nuclear translocation of ERK. During inflammation CCL2 is one of the principal chemokines secreted by astrocytes, endothelial cells, and neurons at the BBB [37] leading to immune cell recruitment to areas prone for infiltration. One previous study investigated the molecular pathways involved in the migratory pattern of DCs versus T cells and dissected the roles of the signaling molecules p38-MAPK and ERK1/2 on cellular trafficking [38]. Interestingly, in response to CCL2 ERK-dependent migratory response in DCs was highlighted, whereas T-cell migration clearly depended on p38-MAPK signaling [38], thus indicating a minor role of ERK inhibition on T cell migration patterns, though thus far the effect of EPE itself has not been tested on brain endothelial cells and their integrity.

Additionally, other pathways besides the ERK cascade might play a more prominent role in Th17 cell pathogenicity since cytokines regulating Th17 differentiation can be mediated through a selective signal transducer and activator of transcription (STAT) transcription factor that functions to regulate lineage-specific gene expression [39,40,41]. It has been reported that STAT3 is required for commitment of naive T cells towards the Th17 developmental pathway, thus suggesting the potential involvement of the STAT3 pathway in mediating CNS inflammatory diseases [39,42].

When investigating the EPE peptide and its functionality, one must consider whether the pharmacology of this specific peptide is suitable for experiments in a rather complex animal model such as EAE. Phosphorylation of the SPS sequence is necessary for binding of ERK to Importin 7 [43]. As a result, ERK is thought to be prevented from binding to Importin 7, a prerequisite enabling ERK to translocate to the nucleus [18,44]. For T lymphocytes, it was shown that interruption of MEK pathway using PD98059 inhibited antigen-induced T cell proliferation [45]. Another study showed that U0126 decreased ERK phosphorylation and restricted T lymphocyte proliferation [21]. All of these inhibitors show beneficial effects upstream of ERK. One future experiment to clarify a clear effect of ERK for autoimmune CNS disorders would be to use a MEK and ERK inhibitor at the same time in mouse models of CNS inflammation. Indeed, we did not address whether this small myristoylated peptide manages to overcome the blood brain barrier (BBB) and thereby has any direct effect in the CNS during EAE. EPE peptide is a competitive inhibitor, therefore the dose-effect curve has to be carefully titrated for each cell type and the bioavailability has to be taken into prospect. Pharmacodynamic and pharmacokinetic properties of brain targeting drugs are still mostly studied with in vivo/in situ methods such as internal carotid perfusion or intracerebral dialysis fibers, and human studies are restricted to post mortem investigations or imaging techniques such as magnetic resonance imaging (MRI) and positron emission tomography (PET) with limited resolution, thus explaining why most of the BBB studies are still restricted to animal models. For our results, more studies are necessary to evaluate the impact of inhibiting pERK in the context of disruption/ crossing of the BBB and to determine the bioavailability of the EPE peptide in the CNS to dissect the poor response in our in vivo model. Possible approaches might be the usage of isolated brain microvessels [46], an easy accessible model maintaining many structural and functional properties of the BBB or the use of the most common and widely used transwell-systems, which are monolayers of endothelial cells grown on a semipermeable membrane separating a luminal and abluminal compartment [47] which is ideal for testing cellular transmigration processes and permeability and binding affinity measurements [48]. Several other in vitro models of the BBB have been developed in recent years, but none of these models replace animal or human studies. Novel possibilities to investigate the bioavailability of certain therapeutics have been published in cancer models where penetration enhancers are coated on biodegradable polymeric nanogels loaded with cytotoxic drugs to determine the chemotherapeutic potential [49]. Alternatively, nanoparticles have been conveniently modified to access a specific promoter region of a cancer’s genome which can be exploited to manipulate the expression of many genes implicated in cancer or also in other diseases [50]. Innovative approaches like these could help to define the impact of the ERK pathway in MS and might improve our poor in vivo response in EAE models, for example, by a sustained release of an inhibitor via nano-drug delivery systems for enhancing the bioavailability of these.

Though EPE peptide shows clear effects in tumor cells and in a xenograft tumor mouse model [18], the response of primary cells to this peptide might differ completely. Furthermore, the relevance of the SPS phosphorylation site needs to be evaluated in more detail in comparison to the phospho-TEY sequence.

## 4. Materials and Methods

### 4.1. Mice

C57BL/6J (B6) and SJL/J mice were purchased from Janvier Labs (Saint-Berthevin Cedex, France). Animal procedures were performed under the supervision of authorized investigators in accordance with the European Union normative for care and use of experimental animals, conducted according to the German Animal Protection Law, and approved by the appropriate state committees for animal welfare (TVA# 23 177-07/G10-1-008, date of approval 8 April 2013).

### 4.2. Induction of Experimental Autoimmune Encephalomyelitis (EAE)

Mice were bred under specifically pathogen-free conditions and kept in-house for experiments in individually ventilated cages. Active EAE in SJL/J mice was induced by subcutaneous four-point immunization with 250 μg PLP peptide and 800 mg H37RA emulsified in Complete Freund’s Adjuvant, followed by two doses of 200 ng pertussis toxin in phosphate buffered saline (PBS) given i.p. at the time of immunization. Active EAE in C57BL/6 mice was induced similarly, with Hooke Kit EK 2110 according to manufacturer’s instructions using MOG_35–55_ peptide instead of the PLP peptide. ERK inhibitory peptide EPE, and MEK inhibitory compound U0126 were diluted in DMSO to a stock concentration of 100 μM and kept at −80 °C. Treatment (15 mg/kg of SCR or EPE or 5 mg/kg of UO126) was given every other day after the clinical assessment at a volume of 50 μL per i.p. administration per mouse, starting two days before immunization. The control mice were given 50 μL DMSO. After induction of EAE, mice were scored daily. Clinical signs of EAE were translated into clinical scores as follows: 0 = no detectable signs of EAE; 1 = complete tail paralysis; 2 = partial hind limb paralysis; 3 = complete bilateral hind limb paralysis; 4 = total paralysis of forelimbs and hind limbs; 5 = death.

### 4.3. Synthesis of Peptide Constructs

The N-myristoylated, C-amidated linear peptides GQLNHILGILGEPEQEDL and GNILSQELPHSGDLQIGL (random control sequence) were manufactured synthetically (Peptide 2.0, Chantilly, VA, USA). Peptide was dissolved to 100 μM in DMSO for further use.

### 4.4. Cell Isolation from Spleen and the Central Nervous System (CNS)

Mice were anesthetized with a 1.5% ketamine solution. The spleen was removed and placed in media substituted with 5% fetal calf serum (FCS) on ice. The rib cage was opened and a needle was inserted into the left ventricle and a small incision was performed in the right atrium. The vascular system of the body circuit was rinsed with 20 mL cold PBS to remove blood cells from the vasculature. The brain and the spinal cord were removed and placed in a tube with 5 mL Iscove's Modified Dulbecco's Medium (IMDM). Brain and spinal cord were cut into small pieces and then digested for 30 min at 37 °C with collagenase IV (Sigma, Taufkirchen, Germany), DNase I (Roche, Mannheim, Germany; Novartis, Nürnberg, Germany) and collagenase/dispase (Roche, Mannheim, Germany), mashed through a 100-μm cell strainer and washed, then the cell pellet was resuspended in 4 mL of a 40% Percoll solution with IMDM, placed on 70% Percoll, and centrifuged for 30 min at 750 g at room temperature (RT) with a very small acceleration and deceleration to generate a gradient. The top layer was discarded and the cell layer of the phase between Percoll at the bottom and IMDM at the top was collected with a 1-mL pipette and washed. Spleens were mashed through a 100-μm cell strainer and washed. To remove erythrocytes, cells were resuspended in lysis buffer (NH_4_Cl (8.29 g/L), KHCO_3_ (1 g/L), NA_2_EDTA (37.2 mg/L)) and washed.

### 4.5. Th17 Cell Culture

Spleens and lymph nodes from B6.2d2 mice (6–10 weeks old) were isolated and sorted naïve as CD4^+^ and CD62L^+^ cells with a purity of >86% of total cells. Th17 cell differentiation was achieved by the addition of 5 ng/mL TGF-β, 10 ng/mL murine recombinant (mr) IL-6, 10 μg/mL anti-IL-4, and 10 μg/mL anti-IFNγ. Cells were kept in cell culture medium for three days and split on day 5. T cells were generally taken on day 3 after a restimulation with PMA and ionomycin and checked for their cytokine expression (Th17 cells: 15–40% IL-17^+^). GM-CSF levels were analyzed by the use of the Mouse GM-CSF Flex Set (BD Cytometric Bead Array (CBA)) according to the manufacturer’s protocol.

### 4.6. Proliferation Assay

CD4^+^ or CD8^+^ T cells were incubated in prewarmed Roswell Park Memorial Institute medium (RPMI) + 1% Hepes. CFSE was added with a final concentration of 2.5 μM and incubated for 10 minutes at 37 °C. Cells were stimulated with plate-bound antibodies against CD3 (3 μg/mL) and CD28 (2.5 μg/mL) in the presence of different concentrations of MEK and ERK inhibitors. Naïve T cells were administered cytokines for the differentiation of Th17 cells. Cells were incubated for at least 72 h prior to analysis of proliferation using flow cytometry. Division index was calculated according to FlowJo defined by the average number of divisions for all cells in the culture [22].

### 4.7. Antibodies

Surface molecules were stained and washed in PBS buffer. Dead cells were stained using Fixable Viability Stain 450 (BD, Bioscience, Heidelberg, Germany) or propidium iodide. For all intracellular and intranuclear staining procedures, the Foxp3/Transcription Factor Staining Buffer Set (eBioscience, Frankfurt, Germany) was used according to the manufacturer’s instructions. The following antibodies were used: CD4-PeCy7 (BD Bioscience, San Jose, CA, USA), IL-17A- AF647 (BD Bioscience), IFNγ- Horizon (BD Bioscience), FoxP3-PE (eBioscience, San Diego, CA, USA) and pERK-APC (eBioscience). Stained cells were measured by FACSCanto II (BD Biosciences) and analyzed with FlowJo software (Treestar, Inc., San Carlos, CA, USA).

### 4.8. Statistics

Statistical analyses were performed using unpaired two-tailed Student's *t* test. Mann-Whitney test was used for nonparametric tests, not assuming Gaussian distributions. Unless otherwise indicated, data are presented as the mean ± standard error of the mean (SEM). *, *p* < 0.05; ns, *p* > 0.05

## 5. Conclusions

In this study, we directly inhibit nuclear ERK translocation by a novel pharmacological approach (EPE peptide), leading to diminished secretion of granulocyte-macrophage colony-stimulating factor (GM-CSF), a cytokine influencing the encephalitogenicity of Th17 cells. Neither the production of the cytokine interleukin (IL)-17 nor the proliferation rate of T cells was affected by the EPE peptide. The in vivo effects of ERK inhibition had only a minor impact on the clinical disease course of EAE. In summary, we conclude that while ERK translocation might promote pathogenicity in Th17 cells in vitro by facilitating GM-CSF production, intervention of this pathway did not translate into beneficial effects in the EAE model.

## Figures and Tables

**Table 1:** Experimental autoimmune encephalomyelitis (EAE) data from all experiments.

Groups	(A) Wildtype (WT) (B) WT + EPE	(A) Wildtype (WT) (B) WT + EPE
*n*	9 per group	8 per group
Incidence	(A) 8/9 (B) 7/9	(A) 5/8 (B) 6/8
Mean day of onset (days ± SEM)	(A) 12.5 ± 0.4 (B) 14.3 ± 0.9	(A) 11.8 ± 0.4 (B) 12 ± 0.4
Mean day of disease maximum (days ± SEM)	(A) 2.9 ± 0.4 (B) 2.6 ± 0.4	(A) 1.8 ± 0.3 (B) 1.3 ± 0.3
Mean maximal score (± SEM)	(A) 2.9 ± 0.1 (B) 2.4 ± 0.3	(A) 2 ± 0.1 (B) 1.7 ± 0.1

## Abbreviations

BBB	Blood brain barrier
CFA	Complete Freund's adjuvant
CFSE	Carboxyfluorescein succinimidyl ester
CNS	Central nervous system
DCs	Dendritic cells
DMSO	Dimethyl sulfoxide
EAE	Experimental autoimmune encephalomyelitis
EPE	Glu-Pro-Glu
ERK	Extracellular signal-regulated kinase
FCS	Fetal calf serum
GM-CSF	Granulocyte-macrophage-colony-stimulating factor
MAP	Mitogen-activated protein
MFI	Mean fluorescence intensity
MOG	Myelin oligodendrocyte glycoprotein
MS	Multiple sclerosis
NTS	Nuclear translocation signal
PBS	Phosphate buffered saline
PLP	Proteolipid protein
PMA	Phorbol 12-myristate 13-acetate
STAT	Signal transducer and activator of transcription
TCR	T cell receptor
WT	Wildtype
